# *“Why not bathe the baby today?”:* A qualitative study of thermal care beliefs and practices in four African sites

**DOI:** 10.1186/s12887-015-0470-0

**Published:** 2015-10-14

**Authors:** Ebunoluwa Aderonke Adejuyigbe, Margaret Helen Bee, Yared Amare, Babatunji Abayomi Omotara, Ruth Buus Iganus, Fatuma Manzi, Donat Dominic Shamba, Jolene Skordis-Worrall, Adetanwa Odebiyi, Zelee Elizabeth Hill

**Affiliations:** Obafemi Awolowo University, Ile-Ife, Nigeria; Institute for Global Health, University College London, 30 Guilford Street, London, WC1N 1EH UK; Consultancy for Social Development, Addis Ababa, Ethiopia; University of Maiduguri, Maiduguri, Nigeria; Ifakara Health Institute, Dar es Saalam, Tanzania

**Keywords:** Thermal care, Wrapping, Delayed bathing, Newborn, Skin to skin care, Qualitative, Africa

## Abstract

**Background:**

Recommendations for care in the first week of a newborn’s life include thermal care practices such as drying and wrapping, skin to skin contact, immediate breastfeeding and delayed bathing. This paper examines beliefs and practices related to neonatal thermal care in three African countries.

**Methods:**

Data were collected in the same way in each site and included 16–20 narrative interviews with recent mothers, eight observations of neonatal bathing, and in-depth interviews with 12–16 mothers, 9–12 grandmothers, eight health workers and 0–12 birth attendants in each site.

**Results:**

We found similarities across sites in relation to understanding the importance of warmth, a lack of opportunities for skin to skin care, beliefs about the importance of several baths per day and beliefs that the Vernix caseosa was related to poor maternal behaviours. There was variation between sites in beliefs and practices around wrapping and drying after delivery, and the timing of the first bath with recent behavior change in some sites. There was near universal early bathing of babies in both Nigerian sites. This was linked to a deep-rooted belief about body odour. When asked about keeping the baby warm, respondents across the sites rarely mentioned recommended thermal care practices, suggesting that these are not perceived as salient.

**Conclusion:**

More effort is needed to promote appropriate thermal care practices both in facilities and at home. Programmers should be aware that changing deep rooted practices, such as early bathing in Nigeria, may take time and should utilize the current beliefs in the importance of neonatal warmth to facilitate behaviour change.

## Background

Neonatal deaths account for 44 % of deaths in children under five, yet neonatal health receives only 4 % of child health investments [1]. Reductions in neonatal mortality rates need to double to reach current targets [2], and progress is particularly slow in sub-Saharan Africa [1]. Improving care in labour, during birth, in the first week of life and for small and sick babies is likely to have the biggest impact on mortality rates [3]. Recommendations for care in the first week of life include improving thermal care practices such as drying and wrapping, skin to skin contact, immediate breastfeeding and delayed bathing [3, 4].

Thermal care is important as newborns are susceptible to hypothermia, even in tropical climates. Newborns have a large body surface area, thin skin, little insulating fat, and limited and easily overwhelmed thermoregulatory mechanisms [5–7]. Newborns lose four times more heat per unit body weight than adults [7]. Without thermal protection newborns are unable to maintain their own body temperature, with preterm babies being particularly at risk [8]. Estimates of hypothermia in African settings are limited to hospital studies, with levels ranging from 44 to 85 %; community studies in Nepal and India have found that hypothermia is near universal at birth [6]. There is a clear biological mechanism for how thermal care interventions could reduce mortality, but high quality studies are lacking [2, 8]. Estimates using the Delphi approach suggest that 20 % of deaths due to prematurity and 10 % of deaths in term babies due to infection could be prevented by improved thermal care practices [2]. In addition the energy expended to maintain body temperature has been linked with reduced head growth in low birth weight babies, which may reflect decreased brain growth at this critical time of development [9].

Formative research collects information on beliefs, attitudes, knowledge and practices, and the contexts that influence these. This gives us an understanding of factors that impede or facilitate appropriate care practices, which is essential for formulating effective intervention strategies that match the local context [3, 10]. Despite the importance of understanding thermal care practices, few studies have explored these issues in depth in sub-Saharan Africa [11, 12], and none has used comparable methods in multiple sites. This paper reports on formative research on thermal care practices in Ethiopia, Nigeria and Tanzania, which, together with nine other countries, account for two-thirds of all neonatal deaths [3]. This study provides information for policy makers in each country and also allows for comparisons between countries to highlight the level of context-specific adaptation that interventions may require.

## Methods

We collected qualitative data on thermal care beliefs practices from one Local Government Area (LGA) in Ekiti State in Southwest Nigeria and two LGAs in Borno State in North East Nigeria, two districts in the Oromiya region of Ethiopia and four districts in Lindi and Mtwara regions of Tanzania.

These sites were selected because of their high neonatal mortality burden, and were diverse in terms of literacy levels, infrastructure, and health care utilization (Table 1). Within study sites, four typical communities were selected to reflect study site diversity in characteristics that could influence newborn care practices such as access to health facilities, ethnicity and geography. In Tanzania, a newborn care trial was being conducted in the study area [13], so data collection was limited to the control areas of this trial. Data were collected during the rainy/cooler season in all sites.

**Table 1:** Study site characteristics

	Borno state Nigeria [32]	Ekiti state Nigeria [32]	Oromiya region Ethiopia [33]	Lindi and Mtwara regions Tanzania [34]
Neonatal mortality rate	43/1000 in North East Zone	39/1000 in South West Zone	40/1000	31/1000 Southern Zone
Female literacy	22 %	93 %	38 %	62 % Lindi and 72 % Mtwara
Any antenatal care	41 %	98 %	39 %	100 %
Facility delivery	17 %	86 %	8 %	52 % Lindi and 59 % Mtwara
Ethnicity	Multi-ethnic	Ekiti group dominates	Oromo and Arsi groups dominate	Multi ethnic
Infrastructure	Poor roads and little electrification	Good roads and widespread electrification	Poor roads and little electrification	Poor roads and little electrification

Data collection included newborn care narratives, observations of bathing and in-depth interviews (IDIs) with recent mothers, grandmothers, fathers, health workers and birth attendants. Data were not collected from birth attendants in Ethiopia as they were rarely used in the study site. The use of multiple methods and a wide range of respondents allowed us to understand thermal care from different perspectives and to corroborate findings. Data were collected as part of a study exploring the potential for emollient therapy in African settings and included specific questions on thermal care. The newborn care narratives collected data on personal experiences and allowed us to understand how events influenced each other. The in-depth interviews collected data on normative behaviors and on the respondents’ experience and beliefs around thermal care practices. The bathing observations aimed to provide a deeper understanding of how practices were actually done and included measuring the length of time the newborn was undressed.

Sample size was based on the concept of saturation sampling, with data collection ending when no new information emerged. This resulted in slightly different sample sizes per site with 16–20 newborn care narratives, eight observations, 12–16 mother IDIs, 9–12 grandmother IDIs, eight health worker IDIs and 0–12 birth attendant IDIs. Community informants identified respondents by word of mouth, or snowball sampling. Mothers for the narrative and IDIs were purposively sampled to ensure a range of maternal ages, parities and sex of child and, where these varied, place of delivery, education level, socio economic status, ethnicity and religion. The characteristics of the narrative women are shown in Table 2, no one refused to participate.

**Table 2:** Characteristics of the women completing narrative interviews

Characteristic	Ekiti, Nigeria	Borno, Nigeria	Ethiopia	Tanzania
Ethnicity	Yoruba: 21	Bura: 10	Oromo: 16	Makonde: 15
		Kanuri: 10		Mwela: 5
Religion				
Christian	21	10	0	3
Muslim	0	10	16	17
Age				
<25	9	4	9	7
26–34	11	10	5	8
≥35	1	6	2	5
Parity				
1	6	1	3	4
2–4	12	9	4	15
≥5	3	10	9	1
Place of delivery				
Home	0	20	20	8
Facility	10	0	0	12
Mission house	11	0	0	0

Data were collected between July and November 2011 and data collection was guided by a study protocol; interview guides were developed by the research team and adapted for each site through pre-testing. Data were collected in the local language by 3–4 trained interviewers in each site. Interviews were conducted in the respondents’ home or workplace and lasted between 30 and 90 min. All interviews were tape-recorded and field notes taken, and these were used to write expanded notes in Microsoft Word, which included verbatim quotes and interviewer observations and reflections [14]. Bathing observations consisted of one person videoing the practice and another taking notes and asking clarifying questions at the end of the observation. Written consent was gained from all participants and ethical clearance was obtained from University College London Research Ethics Committee, Obafemi Awolowo University Teaching Hospital Ethical Review Board, Ekiti State Ministry of Health Review Board, the Research Ethical Review Committee of the Oromia Regional Health Bureau, the University of Maiduguri Teaching Hospital Ethical Committee and Ifakara Health Institute Institutional Review Board.

The site and study coordinators reviewed the expanded notes and tape recordings, and interviewers were provided with feedback on their probing and expanded notes. Regular team meetings were held which included self-reflection and a discussion of methodological issues and emerging themes. Half way through data collection, teams documented key themes in a matrix and modified the guides to ensure missing areas were filled and to remove questions for which saturation had been reached. The study coordinator attended all the training sessions and visited each site during data collection to ensure that comparable methods were being used across the sites.

Formal analysis started with re-reading the transcripts to ensure familiarization. This was followed by group coding of 2–3 interviews to enhance conceptual thinking and rigour [15], and individual coding of the same interview to encourage standardized coding. This initial coding, along with the matrix completed during data collection, was used to develop a codebook and a coding template in NVivo. Sites then coded all interviews using the NVivo template, adding new codes and themes as they emerged. The data were then categorized, organized and interpreted. The NVivo files were sent to the Principal Investigator, who re-coded a sub-set of transcripts and compared and discussed codes with the team. In addition to coding in NVivo, a framework approach using Microsoft Excel was used for the narratives so that themes could be more easily compared and contrasted across and within cases [16]. The video observations were used to provide insight into how practices were performed and to determine the length of exposure during bathing and related activities.

## Results

### Perception of warmth

All respondent groups in all sites understood the need to keep newborns warm, especially if the weather was cold. This was linked to a belief that babies were used to the warmth in the womb and were fragile: *‘When a baby was yet to be born the womb where he was was very hot, that is why if a baby is delivered he wants to be keep warm at all time’* [42 year old Borno mother], and a belief that cold could cause illness *‘Cold can make them have chest pain, the air goes inside the chest of the baby and makes the baby fail to breath properly’* [35 year old Tanzanian mother]. Mothers and grandmothers described cold air entering the body rather than the baby losing body heat.

Respondents were asked how newborns were kept warm in their communities. Themes across the sites were dressing/wrapping the baby well and applying emollients to the skin. Other themes were: bathing the baby with warm water (all sites except Borno); putting the baby on the back (all sites except Ethiopia); delaying the first bath (Ethiopia and Tanzania only) and warming the house in general or during bathing (all sites except Tanzania):

*‘She puts heavy clothes, socks, hat and she warms oil …and rubs on the body of the baby …also she bathes the baby with warm water* [28 year old Tanzanian mother]

*‘We set a fire in order to warm the room and bathe the baby near the fire. A house with a newborn should always stay warm’* [25 year old Ethiopian mother]

During the bathing observations in Ethiopia, we observed that young babies were bathed inside very close to heavily smoking fires.

### Drying and wrapping after birth, and skin-to-skin contact

Data from the narratives show that skin to skin care was almost non-existent in all sites, with very few mothers being given the baby immediately after delivery. The baby was most often placed on a bed, or in Ethiopia, given to relatives to hold. In Ethiopia, babies were usually covered or wrapped, with the birth fluids left to dry naturally or removed with the hands: *‘That wet stuff dries up … it is nothing else but just a wet stuff thus it dries up soon. Therefore the baby was not wiped or anything, he was just wrapped with a cloth’* [22 year old Ethiopian mother]. Reasons given for not wiping the baby in Ethiopia were related to the baby being *‘just blood’* at that time. In Ekiti, data from the narratives suggest that most babies were wrapped immediately after delivery, but few mothers reported on drying. In Borno, immediate wrapping appears less common, with babies either being bathed or cleaned first or placed on the ground until the placenta was delivered. In Tanzania, drying and wrapping was the norm for facility deliveries, but behaviours in the home varied with some babies placed aside until the placenta was delivered. Respondents were not probed on reasons for the timing.

### Timing and temperature of the first bath

The narratives show that bathing occurred soon after delivery in both Nigerian sites but was delayed for several hours or until the next day for most Tanzanian narrative mothers (15/20) and for some Ethiopian narrative mothers (9/15). In Tanzania, delayed bathing was near universal for those who delivered in a facility, but was varied for those who delivered at home (4/8).

In Nigeria, the main reason for the universal early bathing, including at health facilities, was a belief that the birth fluids caused body odour later in life: *‘Hay! You make me laugh…you know the reason why we bathe our newborn is to prevent the child from smelling bad so that when the visitors come they will be so eager to pick the baby and also to prevent the baby from body odour’* [39 year old Borno mother]. In all sites there was a desire for the baby to be clean, neat, comfortable and presentable to visitors and this was a key reason for early bathing when it occurred: *‘We decide to bath the baby because it is very dirty and we can’t leave her with those dirty… it is not good to be seen by other people’* [31 year old Tanzanian mother].

The Vernix was described as dirty in all sites and was linked to poor maternal behaviour such as eating certain foods (all sites), not drinking enough water or not taking certain herbs (all sites), and sex late in pregnancy (Tanzania and the Kanuri group in Borno): *‘If a woman drinks milk which was kept in dirty container or if she eats fatty meat … this white thing would stick on the baby’s skin … when women observe this thing on the newborns skin … they would slur the mother and ask how dare she eat and drink those foods during her pregnancy - negligent’* [35 year old Ethiopian mother]. In Nigeria, the vernix was removed immediately with oil and bathing: *‘My mother in-law used groundnut oil and cotton wool to gently clean the baby’s skin and gave her a bath with warm water, soap and sponge … She had to … completely clean her skin’* [35 year old Borno mother]. In Tanzania and Ethiopia, the presence of an obvious vernix sometimes led to immediate bathing, but for some, wiping was perceived as sufficient to remove the vernix, or it was left to come off gradually over several days. Health workers shared these negative views of the vernix in all sites except Tanzania, where they described the vernix as good for the skin, protecting against infection and helping to keep the newborn warm.

In Tanzania and Ethiopia, delayed bathing appears to be a new practice: *‘I actually wanted my baby to be bathed; all the other children were bathed immediately … I asked them to bathe my baby … the baby comes out with something dirty, he has to be bathed…. these women [who attended her delivery] got education from the health facility … refused to bathe my baby immediately’* [38 year old Ethiopian mother]. Reasons for delayed bathing were health worker advice/action, a fear of cold especially if the baby was born at night and no obvious vernix:

*“He would get cold, therefore he will be immediately wrapped in cloths with the stuff he was delivered with still on him, but if baby is delivered at day time, he will be bathed with lukewarm water right away’* [25 year old Ethiopian mother]

*‘I asked the traditional birth attendant ‘why not bath the baby today?’, she told me in the hospital they … don’t allow you to bath you have to wait up to tomorrow… the traditional birth attendant follows directions which she hears from the hospital’* [34 year old Tanzanian mother]

*‘Since her baby did not have that white thing on his skin, he was bathed later’* [35 year old Ethiopian mother]

Findings from all respondent groups show that in Tanzania and Nigeria warm water was used for the first bath as the baby was perceived as delicate, could get cold, and because warm water gives strength and cold water could shock the baby and make it sick: *‘The baby’s body is very soft and delicate at this tender age and that is why in this community we normally bathe the baby with warm water’* [65 year old Borno grandmother]. In Ethiopia, water temperature varied with some mothers, particularly those in lowland areas, reporting that they used unheated water to get the baby used to cold water or to help the baby feel warm: ‘*If a baby is bathed with cold water, the cold will not get in to her body. She will not feel the cold and will not shiver. But if a baby is bathed with warm water, she will feel cold and shiver when she gets out of the warm water’* [46 year old Ethiopian grandmother]. Other Ethiopian mothers reported using warm water for similar reasons to those given in other sites.

### Subsequent bathing

In all sites newborns were bathed between 2 and 5 times a day and frequent bathing was the cultural norm. Key themes were that bathing was essential for health: *‘Bathing is good…They grow quickly, do not get diseases and gain weight’* [38 year old Ethiopian mother], and important to keep the baby clean, fresh and sweat free and to help them feel comfortable, sleep and grow.

*‘The reason why I normally bathe the baby is for the baby’s well being and good health and also to make the baby comfortable. As I bath the baby very well she will feel refreshed and will sleep very well. The baby will also look clean and neat’* [33 year old Borno mother]

During the bathing observations newborns were exposed for a mean of 23 min in Ethiopia, 11 min in Tanzania, 12 min in Ekiti, and 7 min in Borno. In all sites, the newborns remained undressed after bathing for additional activities, such as cord care (all sites except Ethiopia), massage (Ekiti and Ethiopia), application of emollients (all sites) and application of powder (all sites except Ethiopia).

## Discussion

Many of the thermal care practices were suboptimal. Of particular note was the near universal early bathing of babies in both Nigerian sites, the length of time babies are left undressed during bathing in Ethiopia, and a common belief that bathing with warm water keeps the baby warm.

The link between delayed bathing and body odour later in life has been found in other West African countries [11, 17] but not in East Africa [12, 18–21]. Encouragingly, interventions in Asia have successfully changed bathing practices [22, 23], but results from African trials have been less impressive [13, 24]. Given the regional nature of this deep-rooted belief in the importance of early bathing, behaviour change may be slower in West African countries and programme planners and implementers should be realistic about the time required for behaviour change interventions to have an effect.

Despite significant variation in contexts, we found similarities across sites in relation to understanding the importance of warmth, a lack of opportunities for skin to skin care, multiple baths in a day and negative views of the vernix. An understanding of the importance of newborn warmth has been found in other African studies [8, 11, 12, 25–27], and makes the adoption of appropriate thermal care practices more likely. Skin to skin care was not practised in any of the study sites, even in facilities, and in most cases the baby was physically away from the mother immediately after birth. In Nigeria and Tanzania, respondents mentioned putting the baby on the back to keep them warm, suggesting an understanding that the warmth of the mother can pass to the baby. This understanding may facilitate the adoption of skin to skin care. Only one African study has explored mothers’ actual experiences of skin-to-skin care and identified concerns around disease transmission, harm to the umbilicus, being dirty after birth, and the effect on maternal rest. Mothers liked having immediate access to the baby, feeling close and starting breastfeeding early [28]. More research on the acceptability of skin to skin care is needed.

In countries where facility delivery is common, ensuring that the quality of care in facilities is improved before, or at the same time, as community interventions is important. This would improve the coverage of practices such as skin to skin care for those who delivered at a facility, and may encourage adoption for home births as families may be reluctant to adopt behaviours that are not being carried out at facilities [29].

We found negative perceptions of the vernix in all sites, and the obvious presence of a vernix was a reason for early bathing. In most cases even when bathing was delayed, efforts were made to remove the vernix through wiping. The implication of this for thermal care is unclear as the association between the vernix and thermoregulation is uncertain. Recent evidence suggests that leaving the vernix on enhances skin hydration and acidification which may have an antimicrobial function but more research is needed [30]. In all sites the vernix was linked to poor maternal behaviour including sex in pregnancy in Tanzania and Borno. The link between the vernix and sex has also been reported in other East African countries [18, 20], suggesting that this may be a common belief across the region.

When asked about ways to keep the baby warm, respondents rarely mentioned recommended thermal care practices suggesting that these are not perceived as salient, and that more efforts are needed to promote these behaviours. In Ethiopia respondents mentioned bathing newborns next to the fire, and our observations suggest that this may expose them to heavy smoke, which may increase their risk of respiratory diseases [31].

This study provides useful insights into several key thermal care practices, however, data on breastfeeding, an important thermal care practice [3, 4], were not collected. Other limitations are that there is the potential for reporting bias, especially in those sites where thermal care practices have been promoted by health workers. Data collection in Borno was hampered by Boko Haram activities which limited quality assurance visits by the study coordinator. Data were collected from small geographic areas and the findings may not apply to areas with significant differences in, for example, ethnic groups. The similarity of findings across sites suggests however, that some findings may be widely generalizable. The use of a standard methodology across sites was a strength of the study and a team approach both across and within sites enhanced the rigour of data collection and analysis.

## Conclusion

We found sub optimal thermal care practices in all sites and more effort is needed to promote appropriate practices both in facilities and at home. There were shared beliefs about the importance of thermal care across sites, this understanding makes the adoption of appropriate thermal care practices more likely. Respondents across sites rarely mentioned wrapping and drying after delivery, delayed bathing, or skin to skin care as a means of keeping the baby warm, suggesting that these practices are not yet linked to thermal care or are not salient to families. Reasons for early bathing were also similar across sites, although only in Nigeria did respondents talk of long term consequences. There appear to be recent changes in bathing practices in the Tanzaniana and Ethiopian sites, which is encouraging. Given the deep routed nature of the practice in Nigeria, programmers should be realistic about the speed of behaviour change
